# Hyperbaric Oxygen Ameliorates Bleomycin-Induced Pulmonary Fibrosis in Mice

**DOI:** 10.3389/fmolb.2021.675437

**Published:** 2021-06-04

**Authors:** Yuan Yuan, Yali Li, Guoqiang Qiao, Yilu Zhou, Zijian Xu, Charlotte Hill, Zhenglin Jiang, Yihua Wang

**Affiliations:** ^1^ Department of Neurophysiology and Neuropharmacology, Institute of Special Environmental Medicine and Co-Innovation Center of Neuroregeneration, Nantong University, Nantong, China; ^2^ Biological Sciences, Faculty of Environmental and Life Sciences, University of Southampton, Southampton, United Kingdom; ^3^ Institute for Life Sciences, University of Southampton, Southampton, United Kingdom

**Keywords:** pulmonary fibrosis, hyperbaric oxygen, fibroblast, differentiation, extracellular matrix, HIF-1α

## Abstract

The prevalence of pulmonary fibrosis is increasing with an aging population and its burden is likely to increase following COVID-19, with large financial and medical implications. As approved therapies in pulmonary fibrosis only slow disease progression, there is a significant unmet medical need. Hyperbaric oxygen (HBO) is the inhaling of pure oxygen, under the pressure of greater than one atmosphere absolute, and it has been reported to improve pulmonary function in patients with pulmonary fibrosis. Our recent study suggested that repetitive HBO exposure may affect biological processes in mice lungs such as response to wounding and extracellular matrix. To extend these findings, a bleomycin-induced pulmonary fibrosis mouse model was used to evaluate the effect of repetitive HBO exposure on pulmonary fibrosis. Building on our previous findings, we provide evidence that HBO exposure attenuates bleomycin-induced pulmonary fibrosis in mice. *In vitro*, HBO exposure could reverse, at least partially, transforming growth factor (TGF)-β–induced fibroblast activation, and this effect may be mediated by downregulating TGF-β–induced expression of hypoxia inducible factor (HIF)-1α. These findings support HBO as a potentially life-changing therapy for patients with pulmonary fibrosis, although further research is needed to fully evaluate this.

## Introduction

Pulmonary fibrosis, an interstitial lung disease, is characterized by enhanced deposition and remodeling of the extracellular matrix (ECM), leading to disrupted gas exchange, and ultimately respiratory failure and death (Richeldi et al., 2017). The prevalence of pulmonary fibrosis is increasing with an aging population (Richeldi et al., 2017) and its burden after COVID-19 recovery could be substantial (George et al., 2020). Idiopathic pulmonary fibrosis (IPF), the most common type of progressive fibrotic interstitial lung disease, affects five million people worldwide (Meltzer and Noble, 2008), with a median survival of 3 years (Goodwin and Jenkins, 2016; Richeldi et al., 2017). The current approved therapies for pulmonary fibrosis only slow the disease progression, and as such there is a demand for new treatment options.

Current clinical management of IPF patients includes anti-fibrotic drugs and nonpharmacological support (Richeldi et al., 2017). For patients with advanced disease, reducing symptoms and improving quality of life are required (Zou et al., 2020). Long-term oxygen therapy, with high flow and high concentration of oxygen, is often used to decrease dyspnea and improve exercise tolerance (Koyauchi et al., 2018; Faverio et al., 2019). It is also reported that oxygen supplementation increased exercise capacity for patients with interstitial lung diseases including IPF (Bell et al., 2017; Dowman et al., 2017). Moreover, the benefit of high flow oxygen compared to placebo air was found to improve the quality of life for patients with fibrotic lung disease in a clinical trial (Visca et al., 2018).

Hyperbaric oxygen involves inhaling pure oxygen in a closed chamber pressurized to greater than one atmosphere absolute (ATA). The clinical applications of HBO in ischemic and nonhealing wounds have been reported since the mid-20th century (Lam et al., 2017). An updated list of its applications can be found on the Undersea and Hyperbaric Medical Society Web site (https://www.uhms.org/resources/hbo-indications.html) and have also been reviewed elsewhere (Choudhury, 2018; Kirby, 2019). Interestingly, HBO therapy has been reported to improve pulmonary function in IPF patients (Ma and Du, 2003; Qiu et al., 2013). In another report, HBO exposure reduced radiation-induced side effects including fibrosis in a rat bladder irradiation model (Oscarsson et al., 2017). Mechanistically, our recent study suggested that repetitive HBO exposure may affect biological processes in mice lungs such as response to wounding and ECM (Yuan et al., 2020). To extend these findings, a bleomycin-induced pulmonary fibrosis mouse model was used to evaluate the effect of repetitive HBO exposure on pulmonary fibrosis. Building on our previous report (Yuan et al., 2020), we provide evidence that HBO exposure attenuates bleomycin-induced pulmonary fibrosis in mice. *In vitro*, HBO exposure could reverse, at least partially, transforming growth factor (TGF)-β–induced fibroblast activation. These findings support HBO as a potentially life-changing therapy for patients with pulmonary fibrosis, although further research is needed to fully evaluate this.

## Materials and Methods

### Pathway Enrichment Analysis

The RNA-seq data analyzed were based on our previous study (Yuan et al., 2020) (GSE143348). Briefly, lungs were collected from control mice or HBO-treated mice that were repetitively exposed to 2.5 ATA HBO, 90 min/time, once a day for 11 consecutive days. Control mice were placed in the chamber for the same duration without pure oxygen pressurization. Lung samples were collected on the next day of the last HBO exposure. Total RNA was isolated for library construction, and it was sequenced with the paired-end strategy (2 × 150) on the Illumina NovaSeq 6000 platform following the standard protocols. Enrichment analyses of down-regulated differentially expressed genes (DEGs) were generated by Metascape with default parameters (https://metascape.org/gp/index.html#/main/step1). All significantly enriched Gene Ontology (GO) terms and their *p* values were imported into REVIGO (http://revigo.irb.hr/) to remove redundant GO terms. GO: 0062023 (collagen containing extracellular matrix) and GO: 0031012 (extracellular matrix) gene lists were downloaded from MSigDB Collections (http://www.gsea-msigdb.org/gsea/msigdb/) and converted into corresponding mouse genes. Based on these gene lists, the pathway enrichment score for each sample was calculated by using gene set variation analysis in the GSVA (v1.36.2) package (Hanzelmann et al., 2013).

### Bleomycin-Induced Pulmonary Fibrosis in Mice

Six- to eight-week-old male C57BL/6 mice were purchased from the Experimental Animal Center of Nantong University (institutional license: SYXK(SU)-2012-0030). Mice were maintained under a 12 h light/12 h dark cycle, and normal diet and water were provided *ad libitum* throughout the study. Animal experiments were approved by the Animal Ethics Committee at Nantong University (approval number: 20140901-001).

One dose of 2.0 U/kg of bleomycin (Hisun Pfizer Pharmaceutical Co., Ltd., Zhejiang, China) was intratracheally instilled to induce pulmonary fibrosis in mice. After bleomycin administration, body weights were monitored every third day. According to the previous report, weight loss is an indicator of successful model construction (Vandivort et al., 2016). Mice with a weight loss of less than 5% at day 7 or less than 10% at day 10 post the bleomycin challenge were excluded from further study.

### Hyperbaric Oxygen Exposure of Mice or Cells

A hyperbaric chamber designed for small animal research was used for HBO exposure, as described previously (Yuan et al., 2020). Briefly, after the chamber was flushed with pure oxygen for 5 min, the pressure ramped up to 2.5 ATA (1.5 atm) by inflating 100% oxygen slowly in 5 min, then sustained at 2.5 ATA for 90 min, and finally decompressed slowly in 5 min. The concentrations of carbon dioxide and oxygen were monitored by SDA carbon dioxide and oxygen monitors (Analox, North Yorkshire, England) during the exposure. Bleomycin-challenged mice were randomized into control or HBO-treated group, in which HBO exposure was applied daily from day 7 after intratracheal bleomycin instillation until day 20, and samples were collected at day 21. Mice in the control group were maintained in the normoxia condition throughout the study. Before sample collections, mice were anesthetized with composited anesthetics (257 mM chloral hydrate, 176 mM magnesium sulfate, 36 mM pentobarbital sodium, 14.25% ethanol, and 33.8% propylene glycol).

To treat cells with HBO, a hyperbaric chamber designed for cell culture was used. An embedded circulating water device was used to keep the environmental temperature at 37°C. HBO exposure was applied at 2.5 ATA for 90 min. To maintain the pH of the cell culture medium, the mixed gas with 98% oxygen and 2% carbon dioxide was used to maintain the partial pressure of carbon dioxide at 5 kPa under 2.5 ATA pressure.

### Hematoxylin and Eosin and Masson’s Trichrome Staining

The left lung lobes of the mice were used for morphological examinations. Lungs were fixed with 4% paraformaldehyde for 24 h, dehydrated by gradient ethanol, embedded in paraffin, and sliced 5 μm thick successively. For staining experiments, the tissue sections were dewaxed and rehydrated. For H/E staining, a H/E stain kit (Beyotime Biotechnology, Shanghai, China) was used according to the protocol. For Masson’s trichrome stain, a Masson stain kit (Nanjing Jiancheng Bioengineering Institute, Jiangsu, China) was used following the manufacturer’s instructions. The DM4000B microscope (Leica, Wetzlar, Germany) was used for imaging.

### Ashcroft Score Evaluation

Ashcroft scores were evaluated as previously described (Hubner et al., 2008). To ensure the accuracy of the results, a double-blind strategy was adopted when scoring. Two researchers were asked to score without knowing group information, and the means of the scores for each sample were used for further statistical analysis.

### Hydroxyproline Quantification

Lung tissues were harvested from mice at day 21 after bleomycin administration. Following excision, tissues were immediately flash-frozen in liquid nitrogen. A hydroxyproline assay kit from KeyGEN BioTECH (Jiangsu, China) was used to detected hydroxyproline levels in lungs following the manufacturer’s instructions. Hydroxyproline contents were normalized to the lung tissue mass.

### Cell Culture and Reagents

Human lung fibroblast HFL1 cells were purchased from the Institute of Cell Research (Chinese Academy of Sciences, China) and were cultured in the Nutrient Mixture F-12 Ham (Sigma-Aldrich, MA, United States) cell culture medium containing 10% fetal bovine serum (Gibco, NY, United States) and 1% penicillin/streptomycin. Cells were cultured in a 37°C incubator containing 5% CO_2_. No *mycoplasma* contamination was detected in the cell line used. TGF-β was from PeproTech (NJ, United States).

### Western Blot Analysis

Protein samples from cells or lung tissues were lysed with RIPA buffer (Beyotime Biotechnology, Shanghai, China) containing the protease inhibitor (Meilunbio, Liaoning, China). Primary antibodies were from Cell Signaling Technology (α-SMA, 14968), Sigma-Aldrich (β-actin, A5316), and R&D Systems (HIF-1α, AF 1935). Signals were detected using an ECL detection system with a Tanon 5200 Multi imaging system (Shanghai, China) and evaluated by ImageJ 1.42q software (National Institutes of Health).

### Real-Time qPCR Analysis

Total RNA samples were isolated from cultured cells or lung tissues with the TRIzol reagent (Invitrogen, CA, United States), following the manufacturer’s instructions and quantified using a One Drop OD-1000+ Spectrophotometer (One Drop, Shanghai, China). HiScript II RT SuperMix for qPCR was used for reverse transcriptions (+gDNA wiper) (Vazyme, Jiangsu, China). Universal SYBR qPCR Master Mix was used for qPCR assays (Vazyme, Jiangsu, China). Relative transcript levels of target genes were normalized to β-actin (*ACTB* in human and *Actb* in mouse). Primers for the genes detected were as follows:

Human *ACTA2*-Forward: ACT​GCC​TTG​GTG​TGT​GAC​AA,

Human *ACTA2*-Reverse: CAC​CAT​CAC​CCC​CTG​ATG​TC;

Human *FN1*-Forward: AGG​AAG​CCG​AGG​TTT​TAA​CTG,

Human *FN1*-Reverse: AGG​ACG​CTC​ATA​AGT​GTC​ACC;

Human*COL1A1*-Forward:GAGGGCCAAGACGAAGACATC,

Human*COL1A1*-Reverse:CAG​ATC​ACGTCA​TCGCACAAC;

Human *ACTB*-Forward: GGA​TTC​CTA​TGT​GGG​CGA​CGA,

Human *ACTB*-Reverse: GCG​TAC​AGG​GAT​AGC​ACA​GC;

Mouse *Acta2*-Forward: TCC​CTG​GAG​AAG​AGC​TAC​GAA​C,

Mouse*Acta2*-Reverse:AGG​ACG​TTG​TTA​GCA​TAG​AGA​TCC;

Mouse *Col1a1*-Forward: AGC​ACG​TCT​GGT​TTG​GAG​AG,

Mouse *Col1a1*-Reverse: GAC​ATT​AGG​CGC​AGG​AAG​GT;

Mouse *Fn1*-Forward: CCC​CAA​CTG​GTT​ACC​CTT​CC,

Mouse *Fn1*-Reverse: TGT​CCG​CCT​AAA​GCC​ATG​TT;

Mouse *Actb-*Forward: ACACCCGCCACCAGTTC,

Mouse *Actb-*Reverse: TACAGCCCGGGGAGCAT.

### Statistical Analysis and Repeatability of Experiments

Each experiment was repeated at least twice. Data are presented as mean and standard deviation (s.d.). A two tailed, unpaired, parametric, or nonparametric t-test was used to compare two groups of values, depending on whether the data distribution passed the normality test. One outlier in Ashcroft scores identified by ROUT analysis (*Q* = 1%) was removed from statistical analysis. One-way ANOVA (single-factor analysis of variance) was used to compare more than two groups of data. Two-way ANOVA (two-factors analysis of variance) was applied to analyze the difference of the body weight change curve. GraphPad Prism 8.0 software was used for analysis and *p* < 0.05 was considered as statistically significant.

## Results

### Repetitive Hyperbaric Oxygen Treatments Downregulates Extracellular Matrix Gene Expression in Mouse Lungs

**FIGURE 1:**
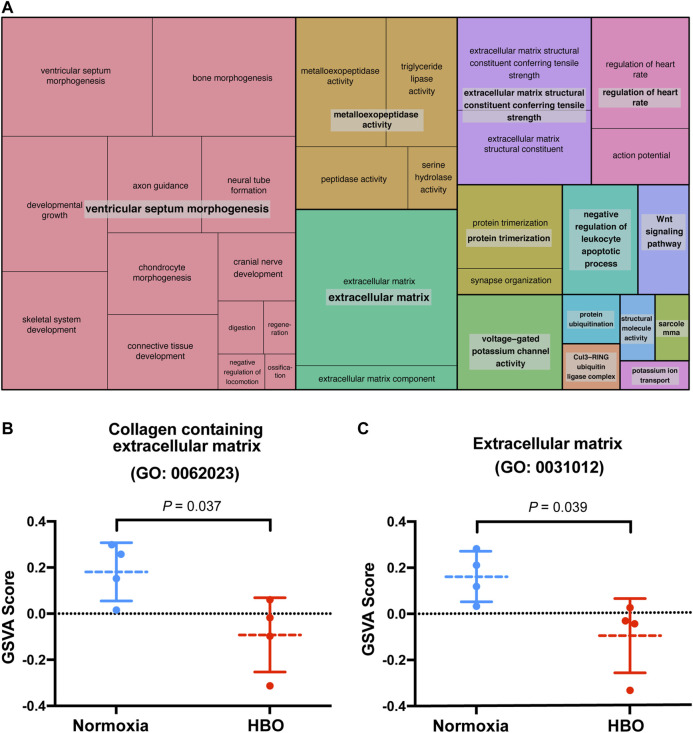
Repetitive HBO treatments downregulate extracellular matrix gene expression in mouse lungs. **(A)** REVIGO TreeMap showing Gene Ontology (GO) analysis of downregulated differentially expressed genes (DEGs) in mice lungs exposed to repetitive HBO (GSE143348). Common colors represent groupings based on parent GO terms, and each rectangle is proportional to the relative enrichment of the GO term compared to the whole genome. Genes with a false discovery rate (FDR) < 0.05 were considered as DEGs. **(B,C)** Graphs showing GSVA scores calculated based on gene lists from GO: 0062023 (collagen containing the extracellular matrix) **(B)** or GO:0031012 (extracellular matrix) **(C)** in HBO-treated and control lungs. Data were analyzed with the unpaired *t*-test. Data are mean ± s.d., with *p* values indicated. *n* = 4 samples per group.

Our previous study suggested that repetitive HBO treaments may affect biological processes in the lungs, such as response to wounding and extracellular matrix (Yuan et al., 2020). We reported that in the down-regulated genes in mice lungs following repetitive HBO exposure (GSE143348), enriched terms for cellular component classification including the “collagen containing extracellular matrix” and “extracellular matrix component,” suggesting that the extracellular matrix may be affected (Yuan et al., 2020). These findings were also reflected using the REVIGO TreeMap, which found the “extracellular matrix” as a GO-enriched term (Figure 1A).

The effect of HBO treatment on ECM genes was further demonstrated through gene set variation analysis (GSVA) using a gene list from GO: 0062023 (collagen containing extracellular matrix); GSVA scores calculated based on this gene list were significantly lower in HBO-treated *vs.* control (normoxia) mice lungs (*p* = 0.037; Figure 1B). Similar results were obtained using another gene list from GO: 0031012 (extracellular matrix) (*p* = 0.039; Figure 1C). Together, these results demonstrate the potential impact of HBO treatment on ECM deposition in mice lungs.

### Repetitive Hyperbaric Oxygen Treatments Attenuate Bleomycin-Induced Pulmonary Fibrosis in Mice

**FIGURE 2:**
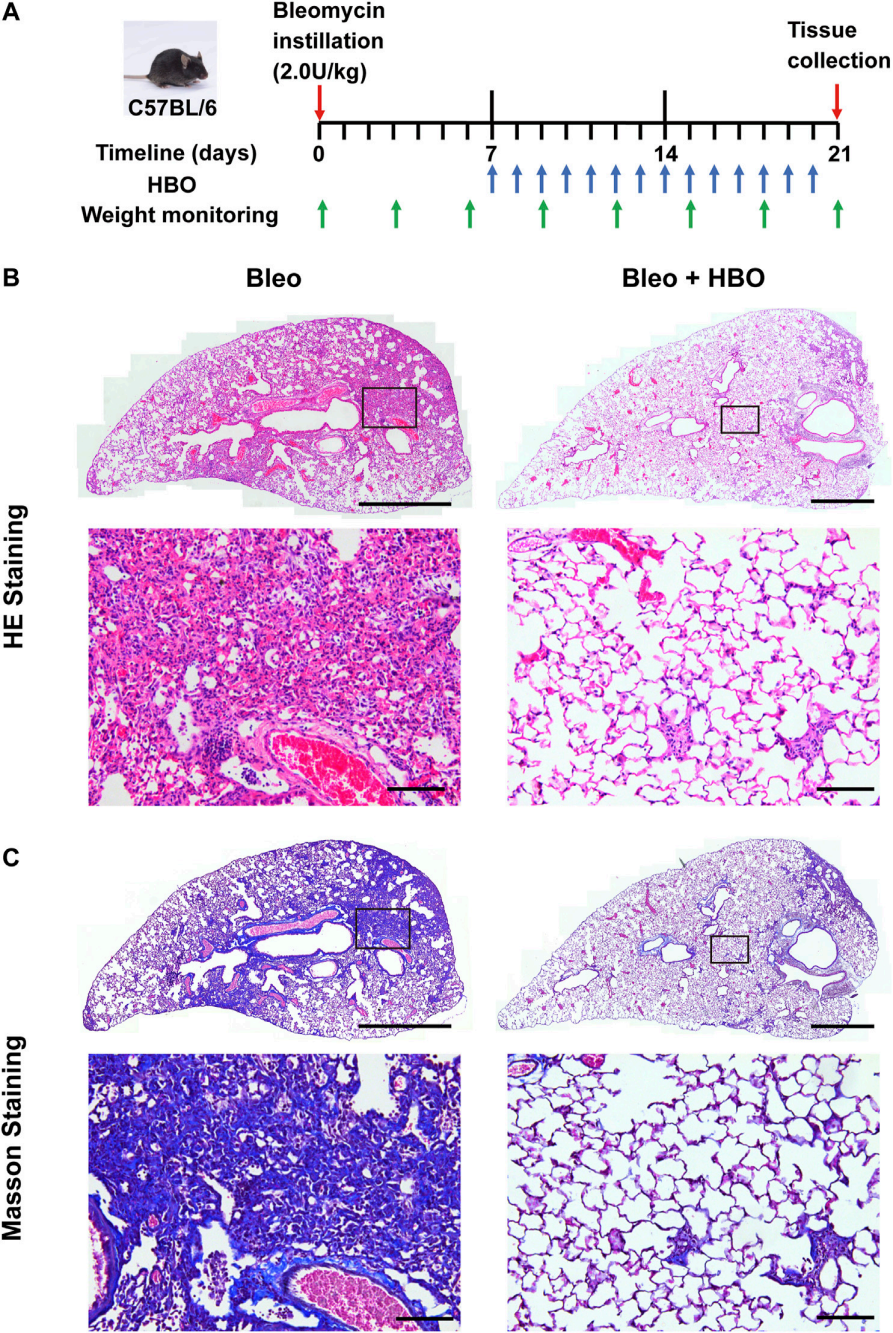
Repetitive HBO treatments reduce the fibrotic area and collagen content in bleomycin-challenged mice lungs. **(A)** Schematic diagram of the experimental procedure (details in *Methods*). **(B,C)** Lung tissues from bleomycin-challenged mice (Bleo) or bleomycin-challenged mice treated with repetitive HBO exposure (Bleo + HBO) were stained with the H/E **(B)** or Masson’s trichrome stain [**(C)**, collagen shown in blue]. In **(B,C)**, top panels show the whole left lung lobes (scale bar: 1 mm) with higher-magnification images in bottom panels (scale bar: 100 μm).

Given the above observation, we next tested whether HBO exposure could affect the development of pulmonary fibrosis, where aberrant ECM deposition is a key feature. To test this hypothesis, bleomycin-induced pulmonary fibrosis in C57BL/6 mice was used (Supplmentary Figure S1). HBO exposure was applied daily from day 7, after intratracheal bleomycin instillation until day 20, one day before sample collections (Figure 2A). Bleomycin-challenged mice showed a clear development of pulmonary fibrosis, with thickened alveoli septae and collagen deposition in the interstitium visualized in the H/E stain (Figure 2B) and Masson’s trichrome stain (Figure 2C). In contrast, fibrotic areas and collagen deposition were markedly reduced in lungs from bleomycin-challenged mice treated with repetitive HBO (Figures 2B,C, right panels).

**FIGURE 3:**
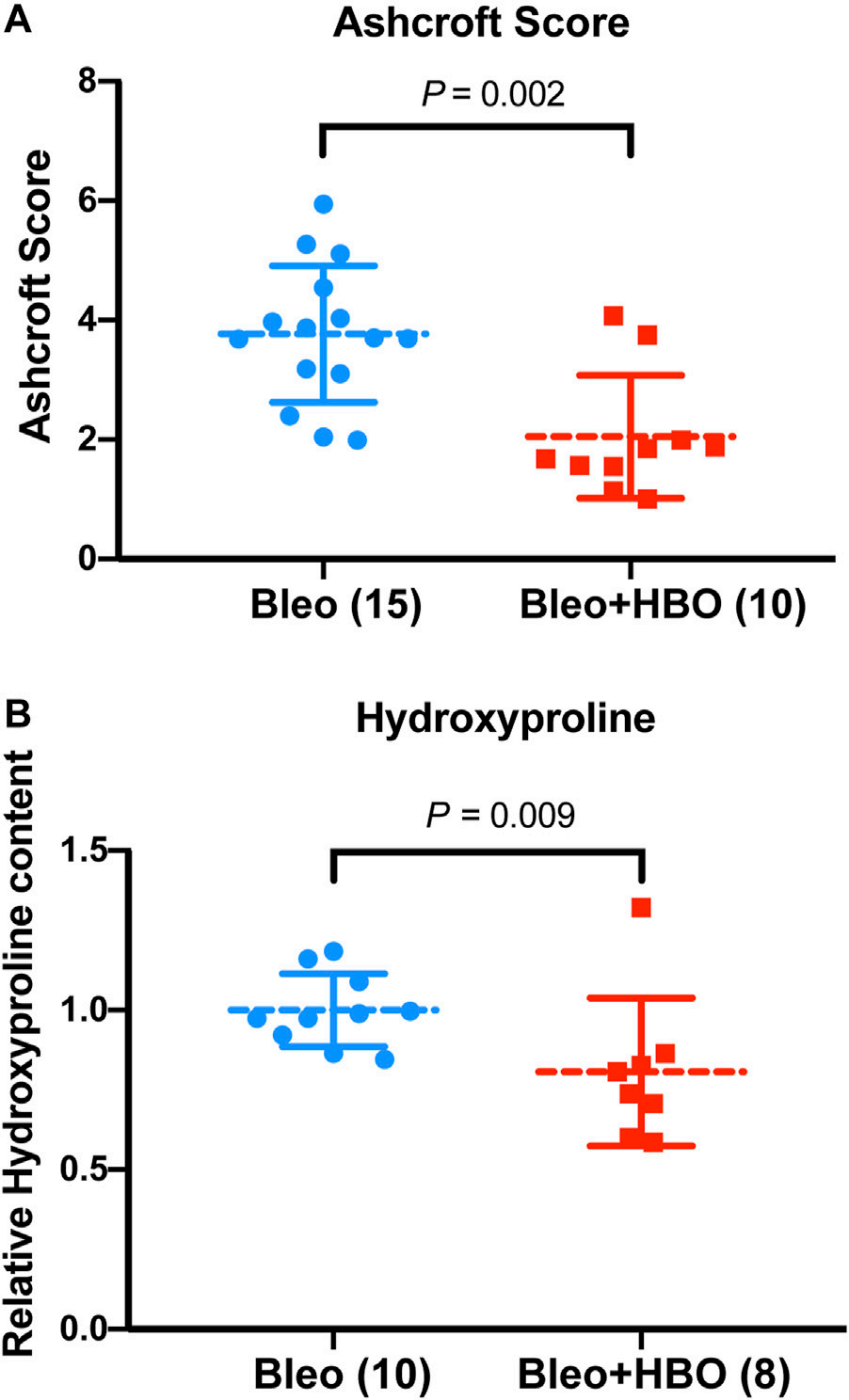
Repetitive HBO treatments attenuate bleomycin-induced pulmonary fibrosis in mice. **(A)** Ashcroft scores in lungs from bleomycin-challenged mice (Bleo) or bleomycin-challenged mice treated with repetitive HBO (Bleo + HBO). Numbers of mice within each group and the *p* value are indicated. **(B)** Graph showing the relative hydroxyproline content in lungs from bleomycin-challenged mice (Bleo) or bleomycin-challenged mice treated with repetitive HBO (Bleo + HBO). Lung tissue mass–normalized hydroxyproline levels in the Bleo group were used to set the baseline value at unity. Data are mean ± s.d., with numbers of mice within each group and the *p* value indicated. Data in **(A)** were analyzed with the unpaired *t*-test. Data in **(B)** were analyzed with the nonparametric *t*-test (Mann–Whitney test).

To quantify the severity of fibrosis, Ashcroft scores were evaluated, and a clear reduction was observed in the lungs from bleomycin-challenged mice treated with repetitive HBO compared to those from bleomycin-challenged mice (*p* = 0.002; Figure 3A). Hydroxyproline is a major component of fibrillar collagen of all types. Consistent with the morphological changes and Ashcroft scores above, the hydroxyproline content was significantly reduced in lungs from bleomycin-challenged mice treated with repetitive HBO (*p* = 0.009; Figure 3B). Effects of HBO on body weight in mice after the bleomycin challenge were minimal (*p* = 0.820; Supplementary Figure S2). Taken together, these data highlight an impact of repetitive HBO exposure on bleomycin-induced pulmonary fibrosis in mice.

### Effect of Repetitive Hyperbaric Oxygen Treatments on Fibroblast Activation and Extracellular Matrix Deposition in Mice Lungs

**FIGURE 4:**
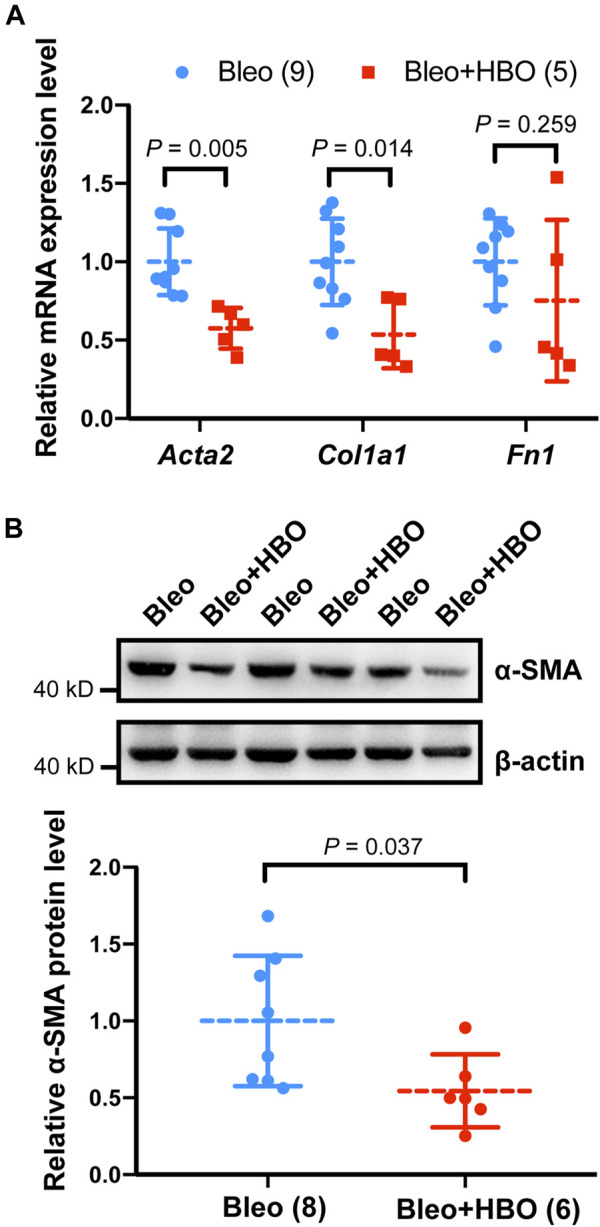
Effects of repetitive HBO treatments on fibroblast activation and ECM deposition in mice lungs. **(A)** Fold change in the mRNA levels of *Acta2* (α-SMA), *Col1a1* (collagen I), and *Fn1* (fibronectin) in the lungs from bleomycin-challenged mice (Bleo) or bleomycin-challenged mice treated with repetitive HBO (Bleo + HBO). *Actb* (β-actin) -normalized mRNA levels in the Bleo group were used to set the baseline value at unity. Data are mean ± s.d., with numbers of mice within each group and the *p* value indicated. **(B)** Protein expression of α-SMA in lungs from bleomycin-challenged mice (Bleo) or bleomycin-challenged mice treated with repetitive HBO (Bleo + HBO). β-actin was used as a loading control. In the graph, β-actin-normalized protein levels in the Bleo group were used to set the baseline value at unity. Data are mean ± s.d., with numbers of mice within each group and the *p* value indicated. Data in **(A)** were analyzed with the unpaired multiple *t*-test. Data in **(B)** were analyzed with the unpaired *t*-test.

We next checked the expression levels of *Acta2* (encoding α-smooth muscle actin, α-SMA, a myofibroblast marker) and other ECM genes, including *Col1a1* (encoding type I collagen) and *Fn1* (encoding fibronectin) in mice lungs. As expected, the mRNA levels of *Acta2* (α-SMA), *Col1a1* (collagen I), and *Fn1* (fibronectin) were significantly increased in the lungs from bleomycin-challenged mice compared to that of the control mice (all *p* values were less than 0.05; Supplementary Figure S3). When the bleomycin-challenged mice were exposed to repetitive HBO treatments, the mRNA level of *Acta2* (α-SMA) and *Col1a1* were both significantly reduced (*p* = 0.005 and 0.014, respectively; Figure 4A). Under the same conditions, the expression of *Fn1* was also decreased, although statistical significance was not reached (*p* = 0.259; Figure 4A). Similar results were obtained when measuring the protein level of α-SMA using western blot (*p* = 0.037; Figure 4B). These results indicate that repetitive HBO exposure could potentially reduce myofibroblast differentiation and activation *in vivo*.

### Effect of Hyperbaric Oxygen Treatment on TGF-β–Induced Fibroblast Activation and HIF-1α Levels in Human Lung Fibroblasts

**FIGURE 5:**
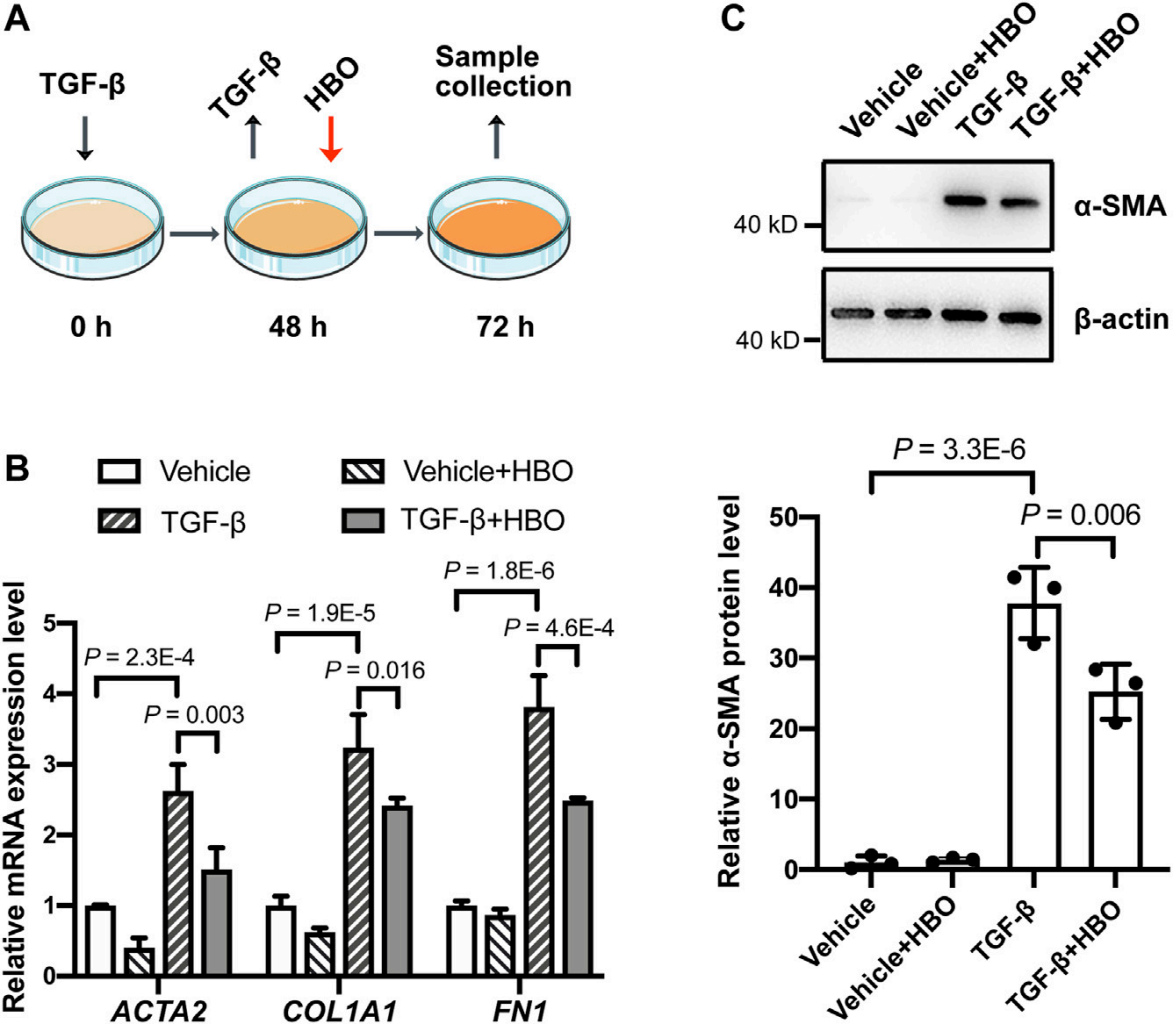
Effects of HBO treatment on TGF-β–induced fibroblast activation in HFL1 cells. **(A)** Schematic diagram of the experimental procedure. In brief, TGF-β (5 ng/ml) was added to HFL1 cells for 48 h to induce fibroblast activation, after which TGF-β was removed, and cells were exposed to 2.5 ATA HBO for 90 min immediately. Samples were collected at 72 h after the beginning of TGF-β treatment. **(B)** The fold change in the mRNA levels of *ACTA2* (α-SMA), *COL1A1* (collagen I), and *FN1*(fibronectin) in HFL1 cells with indicated treatments. *ACTB* (β-actin)-normalized mRNA levels in control cells (vehicle) were used to set the baseline value at unity. **(C)** Protein expression of α-SMA in HFL1 cells with indicated treatments. β-actin was used as a loading control. In the graph, β-actin–normalized protein levels in control cells (vehicle) were used to set the baseline value at unity. Data in **(B,C)** are mean ± s.d., with *p* values indicated. *n* = 3 samples each group. Data were analyzed with one-way ANOVA.

To validate the findings *in vitro*, the effects of HBO treatment on TGF-β–induced fibroblast activation in human lung fibroblasts HFL1 were examined (Figure 5A). After incubating HFL1 cells with TGF-β for 48 h, the mRNA levels of *ACTA2* (α-SMA), *COL1A1* (collagen I), and *FN1*(fibronectin) were all induced in HFL1 cells compared to that of control cells (all *p* values were less than 0.05; Supplementary Figure S4), indicating fibroblasts were activated. TGF-β was then removed and HBO exposure was applied to HFL1 cells for 90 min (Figure 5A). At 72 h after TGF-β treatment, *ACTA2* (α-SMA), *COL1A1,* and *FN1* sustained at high expression levels in TGF-β-treated groups compared to that of controls (all *p* values were less than 0.05; Figure 5B). In TGF-β–treated cells, the mRNA levels of *ACTA2* (α-SMA), *COL1A1,* and *FN1* were significantly reduced when exposed to HBO (all *p* values were less than 0.05; Figure 5B). In the absence of TGF-β, the mRNA levels of *ACTA2* (α-SMA), *COL1A1,* and *FN1* were also decreased when exposed to HBO, although statistical significance was not reached (Figure 5B). Similar results were obtained when measuring the protein level of α-SMA using western blot (Figure 5C). In addition, to test if HBO treatment could block TGF-β–induced fibroblast differentiation, HBO exposure was applied immediately following TGF-β treatment (Supplementary Figure S5A). Q-PCR showed that this treatment could also reduce TGF-β–induced *ACTA2* (α-SMA) mRNA levels in HFL1 cells (*p* = 0.043) (Supplementary Figure S5B). These results suggested that HBO exposure could reverse and block, at least partially, TGF-β–induced fibroblast activation.

**FIGURE 6:**
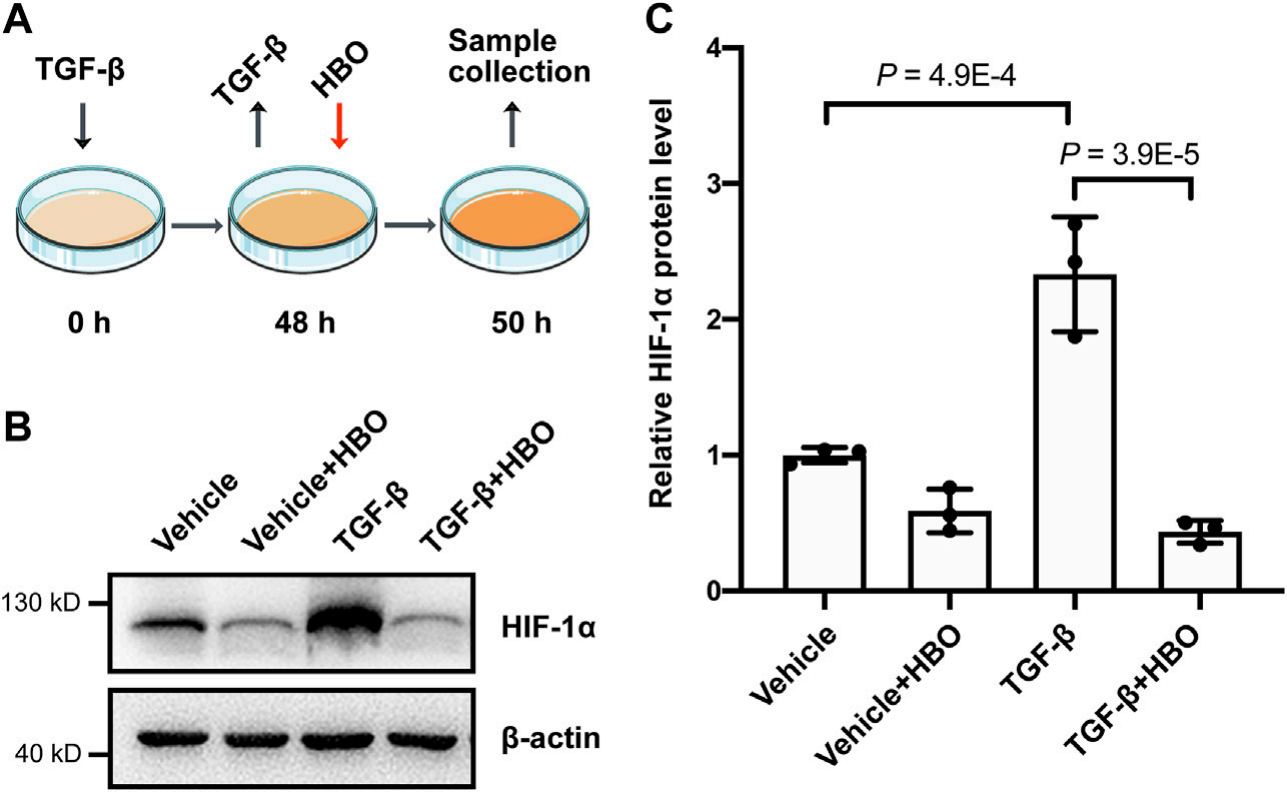
Effects of HBO treatment on TGF-β–induced HIF-1α expression in HFL1 cells. **(A)** Schematic diagram of the experimental procedure. In brief, TGF-β (5 ng/ml) was added to HFL1 cells for 48 h to induce fibroblast activation, after which TGF-β was removed, and followed 2.5 ATA HBO exposure for 90 min immediately. Samples were collected at the end of HBO exposure. **(B)** Protein expression of HIF-1α in HFL1 cells with indicated treatments. β-actin was used as a loading control. **(C)** Fold change in the protein level of HIF-1α in HFL1 cells with indicated treatments. β-actin–normalized protein levels in control cells (vehicle) were used to set the baseline value at unity. Data are mean ± s.d., with *p* values indicated. *n* = 3 samples each group. Data were analyzed with one-way ANOVA.

Finally, we checked the effects of HBO treatment on HIF-1α levels following TGF-β treatment in human lung fibroblasts (Figure 6A). In consistence with previous studies (Yamazaki et al., 2017; Senavirathna et al., 2020), TGF-β treatment significantly upregulated the protein levels of HIF-1α in HFL1 (*p* = 4.9E-4; Figures 6B,C). As expected, HBO exposure dramatically reduced TGF-β–induced HIF-1α protein expression (*p* = 3.9E-5; Figures 6B,C). In addition, we were able to show that HBO exposure can also block TGF-β–induced HIF-1α levels in HFL1 (Supplementary Figures S5C–E).

## Discussion

Pulmonary fibrosis is a chronic, progressive lung disease with limited therapeutic options (Richeldi et al., 2017). In this study, we utilized an animal model and assessment methods for pulmonary fibrosis recommended by the American Thoracic Society (Jenkins et al., 2017). We report that repetitive HBO exposure attenuates bleomycin-induced pulmonary fibrosis in mice, and that HBO exposure, both *in vivo* and *in vitro*, inhibits fibroblast activation and ECM production. HBO therapy is generally very safe (Camporesi, 2014; Hadanny et al., 2016; Hadanny et al., 2019) and has been used in a variety of clinical practices (Choudhury, 2018; Kirby, 2019). Together with earlier reports indicating an improvement of pulmonary function in IPF patients following HBO therapy (Ma and Du, 2003; Qiu et al., 2013), our findings support HBO as a potential therapy for patients with pulmonary fibrosis.

As a master regulator of fibroblast activation, it was previously reported that in human lung fibroblasts, TGF-β upregulates the protein levels of HIF-1α and synergistically increases the expression of myofibroblast markers and ECM genes (Senavirathna et al., 2020). In addition, TGF-β–induced fibroblast activation is suppressed by HIF-1α inhibition in human lung fibroblasts (Yamazaki et al., 2017). With evidence in this study showing the ability of HBO to prevent and reverse TGF-β–induced HIF-1α expression, we propose that HBO exposure affects TGF-β–induced fibroblast activation by modulating the expression of HIF-1α.

In addition to the effect of counteracting the upregulation of HIF-1α induced by TGF-β, HBO is reported to reduce HIF-1α levels through alleviating tissue hypoxia, in a similar manner to multiple ischemic conditions, injuries, and inflammatory conditions (Li et al., 2005; Calvert et al., 2006; Sun et al., 2008; Bai et al., 2009; Zhou et al., 2013). Hypoxia is a hallmark of pulmonary fibrosis. Previously, studies have shown that the hypoxia signaling pathway was activated in IPF patients (Tzouvelekis et al., 2007; Ueno et al., 2011; Xie et al., 2013; Qian et al., 2015; Kusko et al., 2016; Philip et al., 2017; Yamazaki et al., 2017; Burman et al., 2018; Aquino-Galvez et al., 2019), Further, chronic exposure to hypoxic conditions can increase the severity of bleomycin-induced pulmonary fibrosis in murine models (Braun et al., 2018; Burman et al., 2018; Gille et al., 2018). Furthermore, inhibition of HIF-1α, directly or indirectly, alleviates pulmonary fibrosis in the bleomycin-induced model (Yamazaki et al., 2017; Goodwin et al., 2018; Strowitzki et al., 2019; Kseibati et al., 2020). Also, hypoxia induced fibroblast differentiation directly and this effect depended on HIF-1α (Robinson et al., 2012; Lv et al., 2018). HBO is an effective way of oxygenating hypoxic tissues through increasing the dissolved oxygen in plasma and amplifying oxygen diffusion distance under higher pressure. Its effect on alleviating tissue hypoxia has been confirmed in solid tumors (Kinoshita et al., 2000; Beppu et al., 2002; Thews and Vaupel, 2016) and focal cerebral ischemia tissue (Sun et al., 2008).

Given both hypoxia and TGF-β signaling pathways are activated in pulmonary fibrosis, HBO may inhibit HIF-1α expression induced by both hypoxia and TGF-β. Previous studies suggested that the effect on alleviating tissue hypoxia by HBO can only maintain for a certain time (Kinoshita et al., 2000; Beppu et al., 2002; Thews and Vaupel, 2016), suggesting that repetitive HBO exposure is required. Future studies are needed to optimize the protocol for the clinical application of applying HBO as a therapy for pulmonary fibrosis.

In summary, this study provides evidence that HBO exposure attenuates bleomycin-induced pulmonary fibrosis *in vivo* and TGF-β–induced fibroblast activation *in vitro*. Mechanistically, this effect may be mediated by downregulating TGF-β–induced expression of HIF-1α. These findings support HBO as a potential life-changing therapy for patients with pulmonary fibrosis, although further research is needed to fully evaluate this.

## Data Availability

Publicly available datasets were analyzed in this study. These data can be found in the Gene Expression Omnibus (GEO) database: accession code GSE143348.
