# Supplementary material for: Hyperbaric Oxygen Ameliorates Bleomycin-Induced Pulmonary Fibrosis in Mice
Source: Front Mol Biosci. 2021 Jun 4;8:675437. doi: 10.3389/fmolb.2021.675437 (PMC8211992; doi:10.3389/fmolb.2021.675437)
Supplement: Supplementary file 1 [file DataSheet1.pdf]

Supplementary Material

Supplementary Figures

Supplementary Figure 1. Bleomycin treatment induces pulmonary fibrosis in mice.

Supplementary Figure 2. Body weight changes in bleomycin-challenged mice (Bleo) or bleomycin-challenged mice treated with repetitive HBO (Bleo + HBO).

Supplementary Figure 3. Effects of bleomycin on fibroblast activation and ECM deposition in mice lungs.

Supplementary Figure 4. Effects of TGF-β on fibroblast activation in HFL1 cells.

Supplementary Figure 5. Effects of HBO treatment on TGF-β-induced fibroblast differentiation and HIF-1α expression.

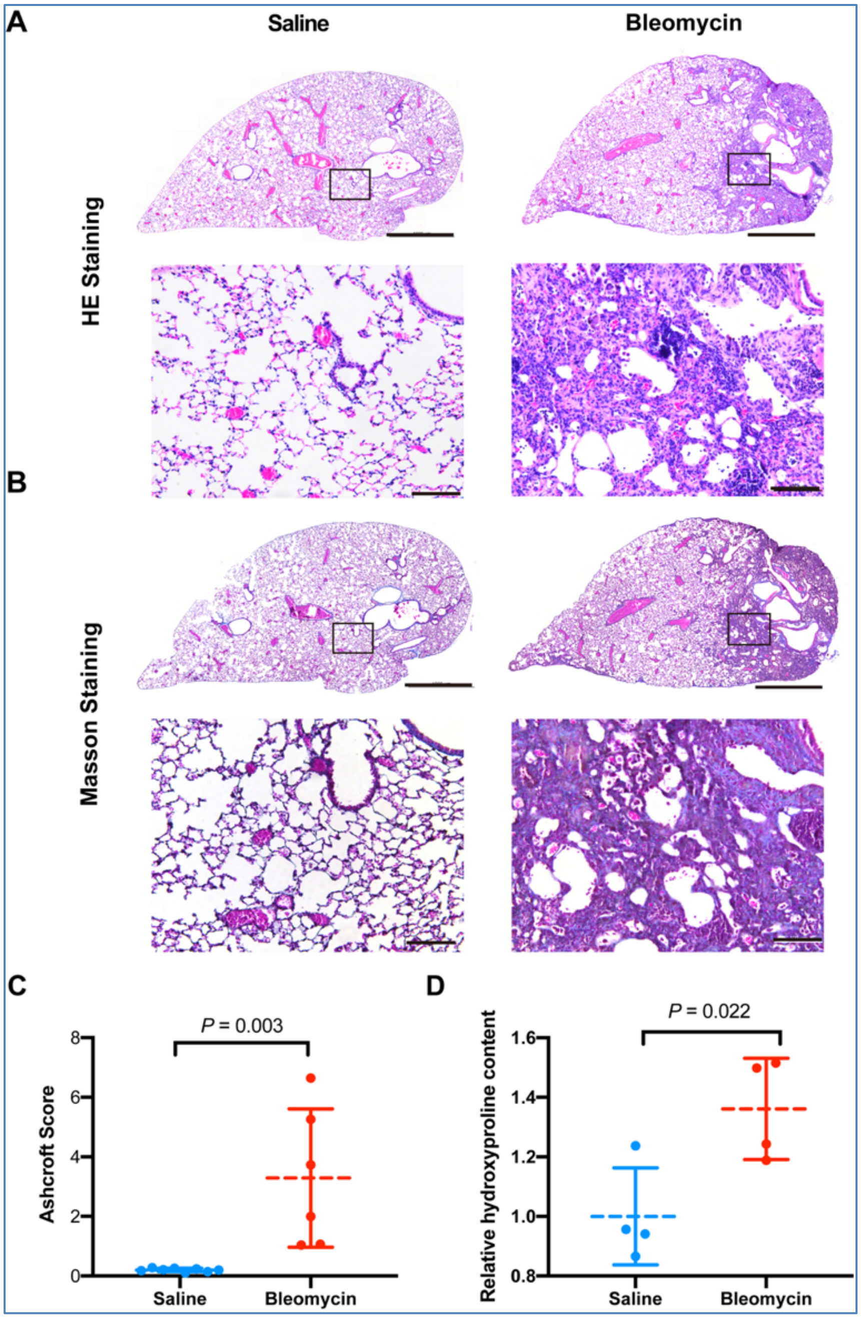

Supplementary Figure 1. Bleomycin treatment induces pulmonary fibrosis in mice. (A and **B)** Lungs from saline-treated (Saline) or bleomycin-challenged mice (Bleomycin) collected at day 21 post instillation were stained with H/E **(A)** or Masson's trichrome stain **(B**, collagen shown in blue**)**. Top panels show the whole left lung lobe (scale bar: 1 mm) with higher-magnification images in bottom panels (scale bar: 100 μm). **(C)** Graph showing Ashcroft scores in lungs from saline-treated (Saline) or bleomycin-challenged (Bleomycin) mice. **(D)** Graph showing relative hydroxyproline content in lungs from saline-treated (Saline) or bleomycin-challenged (Bleomycin) mice. Lung tissue mass-normalised hydroxyproline levels in saline group were used to set the baseline value at unity. Data are mean ± s.d., with *P* values analysed with unpaired *t*-test.

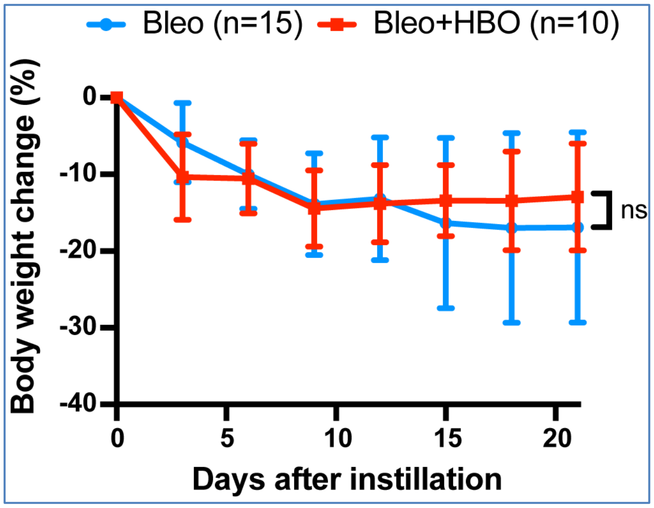

Supplementary Figure 2. Body weight changes in bleomycin-challenged mice (Bleo) or bleomycin-challenged mice treated with repetitive HBO (Bleo + HBO). Body weights were measured every third day. A Two-way ANOVA test with repeated measure data was used to analyse the difference with no significant (ns) difference identified. Data are mean ± s.d., with numbers of mice within each group indicated.

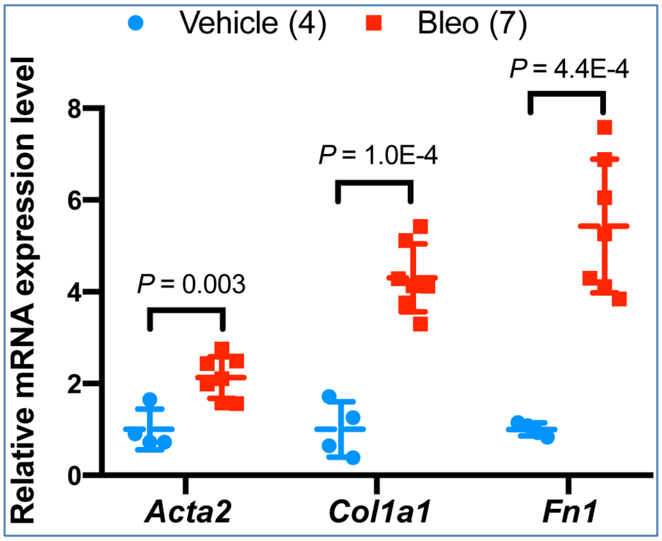

Supplementary Figure 3. Effects of bleomycin on fibroblast activation and ECM deposition in mice lungs. Fold change in the mRNA levels of *Acta2* (α-SMA), *Col1a1* (collagen I) and *Fn1* (fibronectin) in lungs from control (Vehicle) or bleomycin-challenged (Bleo) mice. *Actb* (β-actin)-normalised mRNA levels in control mice lungs were used to set the baseline value at unity. Data are mean ± s.d., with numbers of mice within each group and *P* values indicated. Data were analysed with multiple *t*-test.

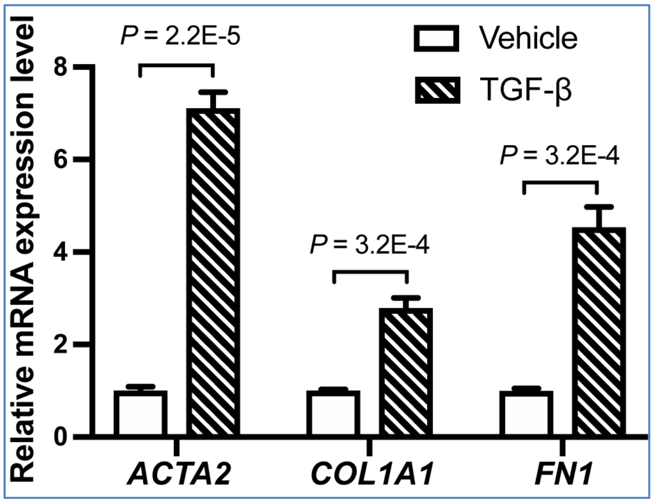

Supplementary Figure 4. Effects of TGF-β on fibroblast activation in HFL1 cells. Fold change in the mRNA levels of *ACTA2* (α-SMA), *COL1A1* (collagen I) and *FN1*(fibronectin) in HFL1 cells with indicated treatments. *ACTB* (β-actin)-normalised mRNA levels in control cells (Vehicle) were used to set the baseline value at unity. Data are mean ± s.d., with *P* values indicated. n = 3 samples each group. Data were analysed with multiple *t*-test.

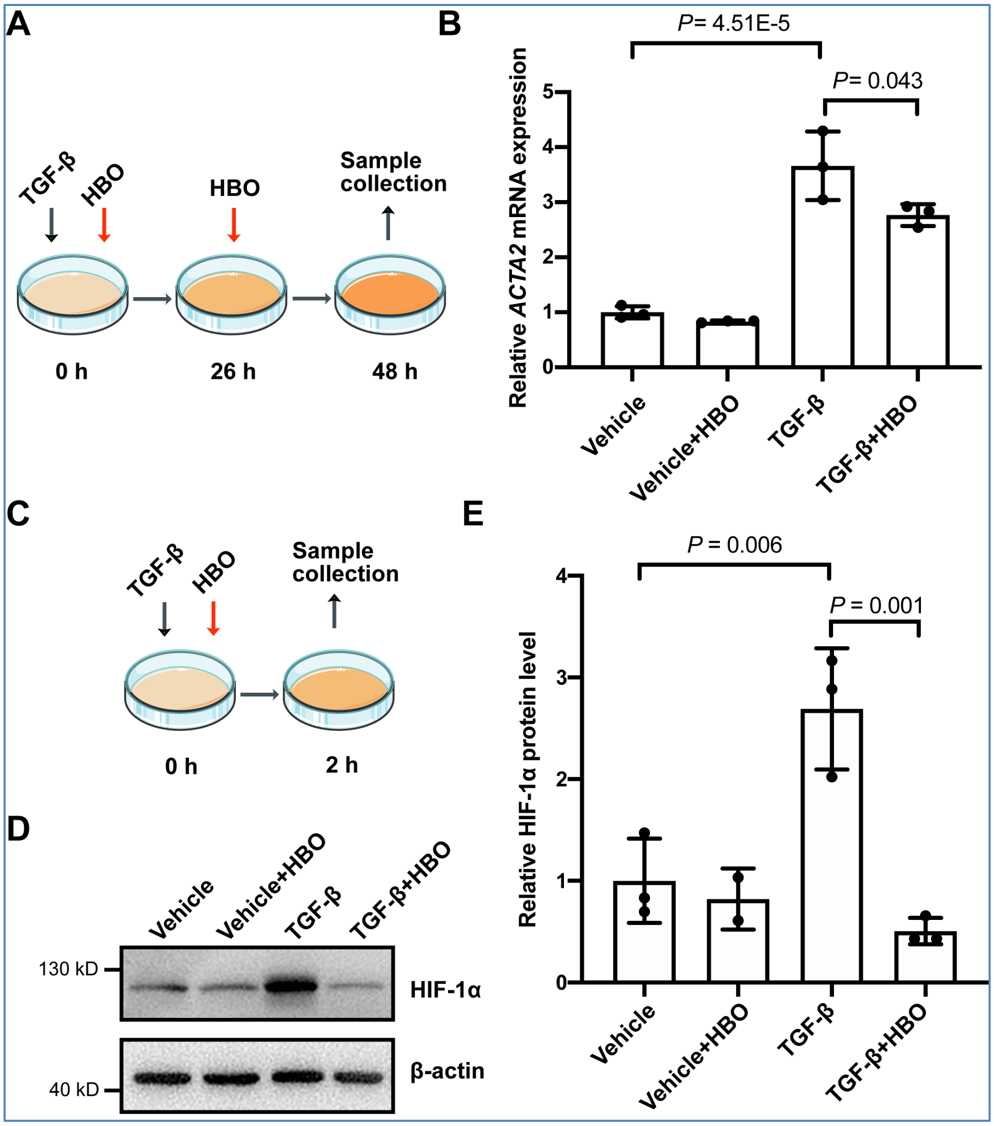

Supplementary Figure 5. Effects of HBO treatment on TGF-β-induced fibroblast differentiation and HIF-1α expression. (A) Schematic diagram of the experimental procedure. In brief, TGF-β (5 ng/mL) was added to HFL1 cells for 48 hours to induce fibroblast activation. At the beginning of TGF-β treatment, HFL1 cells were exposed to 2.5 ATA HBO for 90 minutes immediately. The HBO exposure was repeated for another time with a 24-hours interval (about 26 h post TGF-β treatment). Samples were collected at 48 hours after the beginning of TGF-β treatment. **(B)** Fold change in the mRNA levels of *ACTA2* (α-SMA) in HFL1 cells with indicated treatments. *ACTB* (β-actin)-normalised mRNA levels in control cells (Vehicle) were used to set the baseline value at unity. **(C)** Schematic diagram of the experimental procedure. In brief, HFL1 cells were exposed to 2.5 ATA HBO for 90 minutes right after TGF-β (5 ng/mL) treatment. At the end of HBO exposure, samples were collected immediately. **(D)** Protein expression of HIF-1α in HFL1 cells with indicated treatments. β-actin was used as a loading control. **(E)** Fold change in the protein level of HIF-1α in HFL1 cells with indicated treatments. In the graph, β-actin-normalised protein levels in control cells (Vehicle) were used to set the baseline value at unity. Data in **(B and E)** are mean ± s.d., with *P* values indicated. Data were analysed with one-way ANOVA.
